# Supplementary material for: Machine learning-based predictive modeling of angina pectoris in an elderly community-dwelling population: Results from the PoCOsteo study
Source: PLoS One. 2025 Aug 5;20(8):e0329023. doi: 10.1371/journal.pone.0329023 (PMC12324099; doi:10.1371/journal.pone.0329023)
Supplement: Attachment — Submitted filename: Response to Reviewers.docx [file pone.0329023.s002.docx]

**Reviewer #1**

We thank the reviewer for their thoughtful and constructive comments, which have greatly contributed to enhancing the quality and clarity of our manuscript. In response to each of the points raised, we have made several revisions including an expanded discussion on OSA, additional details on the validation of our measurement tools, clearer descriptions of our study design and sample demographics, comparative discussions of our findings with previous research, and a more concise conclusion that emphasizes the clinical and public health implications of our work. We have outlined our responses point‐by‐point below.

**Comment 1**: The manuscript successfully identifies OSA as a significant public health issue associated with cardiovascular comorbidities such as hypertension, diabetes, and myocardial infarction. However, the study could benefit from explicitly linking these findings to the broader epidemiological context of sleep apnea in the target population. The authors can include a brief discussion on the prevalence of OSA and its associated cardiovascular risks in your study population, possibly using existing regional data as a reference.

**Response 1**: We thank the reviewer for this valuable suggestion. In the revised manuscript, we have expanded the discussion by including additional context on the prevalence of OSA in our study population, drawing on regional and national epidemiological data (paragraph starting on line 526). This section now further emphasizes the high burden of OSA and its established association with cardiovascular risks.

**Comment 2**: The use of standardized tools like the RAQ is commendable, but it would be valuable to confirm whether these tools were validated for your specific population or if local adaptations were made. Additionally, while the measurement of grip force and anthropometric variables is appropriate, it would enhance the methodology section to explicitly mention any validation studies or interobserver reliability assessments for these measures. The authors may cite any validated scales or tools used in this study and provide references to their validation studies.

**Response 2**: Thank you for this comment regarding the validity and reliability of the mentioned measures. Yes, the Persian translation of RAQ has been used, and it has been previously validated by Najafi-Ghezeljeh and has been confirmed as a reliable tool (Line 172). Additionally, we have now included details on the protocols used for grip force and anthropometric measurements, along with references to established validation studies and reliability assessments (Line 200). Thank you for helping us improve our manuscript.

**Comment 3**: The manuscript provides a comprehensive overview of data collection procedures, including medical history, physical examinations, and questionnaires. However, the sample size is not clearly specified. To ensure generalizability, it would be helpful to clarify whether this study was part of a larger prospective or retrospective cohort and, if applicable, provide demographic details about your participants (e.g., age, gender distribution, education level, etc.). The authors are suggested to specify whether this study is cross-sectional or part of a larger longitudinal investigation. If applicable, include basic demographics of the study population to enhance external validity.

**Response 3**: Thank you for your insightful comment regarding the need to clarify the sample size and demographic details of our study. In response, we have revised the manuscript to explicitly state the overall sample size (line 139), describe the study design (Line 137 and paragraph starting on line 128), and provide basic demographic information for our participants (line 140 and line 380). Specifically, we have now clarified that the data analyzed in this study has been extracted from only one time point of a larger prospective cohort investigation. Therefore, the current study is designed as a cross-sectional investigation (Line 137 and paragraph starting on line 128). We appreciate your suggestion, as these additions help to improve the clarity and generalizability of our research.

**Comment 4**: The presentation of results is clear and organized, with mean values provided for key variables like blood pressure, grip force, and anthropometric measurements. However, some of the p-values (e.g., <0.001) are not interpreted in the context of other studies. This could limit readers' understanding of the statistical significance relative to previous findings. The authors should provide a brief discussion of how the observed results compare to those in prior studies on OSA and cardiovascular risk factors. For example, reference studies that report similar p-values for blood pressure measurements or grip force in sleep apnea patients.

**Response 4**: We thank the reviewer for highlighting this point. In our revised manuscript, we have added a brief discussion comparing our findings for key variables with those reported in previous studies examining similar associations (paragraphs starting on lines 504, 517, and 526).

**Comment 5**: While the manuscript provides valuable insights into the relationship between OSA and cardiovascular comorbidities, there is no explicit discussion section to contextualize these findings within the broader scientific literature. A brief discussion linking your results to prior research would greatly enhance the manuscript's impact. The authors may include a discussion where you compare your findings to existing studies on sleep apnea and its complications, focusing on similarities, differences, and potential mechanisms.

**Response 5**: We appreciate the reviewer’s suggestion to enhance the manuscript’s impact by integrating our findings with the broader literature. In response, we have now incorporated a discussion section that contextualizes our results within existing research. These additions include comparisons with previous studies and explore potential underlying mechanisms linking OSA to cardiovascular comorbidities (paragraphs starting on lines 526 and 545).

**Comment 6**: The conclusion is concise but could be improved by summarizing the key findings without repeating previous results or data. Additionally, it would be beneficial to highlight the implications of your study for clinical practice and public health strategies. In the conclusion, the authors may explicitly state how your findings can inform clinical decisions, such as recommending polysomnography for high-risk patients or implementing targeted intervention programs.

**Response 6**: Thank you for your constructive feedback regarding the conclusion section (paragraph starting on line 573). Accordingly, we have now revised the conclusion to provide a concise summary of our key findings without repetition and to clearly outline the clinical and public health implications of our work. In the revised conclusion, we emphasize how the identified predictors can inform targeted intervention programs and support clinical decisions, such as recommending additional diagnostic tools for high-risk patients.

**Reviewer #2**

We thank the reviewer for their comprehensive and insightful comments, which have significantly contributed to clarifying the methods and strengthening the discussion in our manuscript. In response to the points raised, we have provided detailed clarifications on our data preprocessing steps, including the handling of missing values and outliers, as well as the normalization and scaling techniques applied to our predictor variables. We have revised the abstract for improved coherence by explicitly highlighting numerical performance improvements, and we have expanded the manuscript to better address the complexity of our models, and elaborate on the hyperparameter optimization process. Finally, additional explanations regarding our methodological choices have been integrated to enhance the overall clarity and rigor of our study. We detail our point‐by‐point responses below.

**Comment 1**: How were missing values and outliers in the medical records handled prior to model training?

**Response 1**: Thank you for pointing out this issue. Changes were made on line 36 and line 238. Preprocessing in this analysis handled missing values only with the SimpleImputer function from the scikit-learn library, replacing each missing value with the average of each column (*strategy=’mean’*) for quantitative variables and most frequent values for the qualitative variables (*strategy=’most_frequent’*). As for the scale of each variable, the StandardScaler function was used, including both high and low extremes (Line 241).

**Comment 2**: Were any normalization or scaling techniques applied to the predictor variables?

**Response 2**: Thank you for your question. Yes, it was. Changes are now made on lines 36 and 243. Scaling was applied to the predictor variables using the StandardScaler function from the Scikit-learn library (Line 241).

**Comment 3**: The abstract is not coherent. It would be good if authors can write a sentence describing numerical results and improvement over other methods.

**Response 3**: Thank you for your constructive feedback. We have revised the abstract for improved coherence and included an additional sentence that explicitly describes the numerical performance results (Line 48). This sentence highlights the specific improvements observed in our best-performing models compared to the others.

**Comment 4**: The complexity of the proposed model and the model parameter uncertainty are not enough mentioned.

**Response 4**: Thank you for pointing this issue out. Editions are now made on line 40 and line 53.

**Comment 5**: Discussions" section should be added in a more highlighting, argumentative way. The author should analysis the reason why the tested results is achieved.

**Response 5**: Thank you for your valuable feedback. We have revised our Discussion section to be more argumentative and highlighted the potential reasons behind the achieved results (Line 54). We now elaborate on how the inherent characteristics of the top-performing models (linear discriminant analysis, random forest, and logistic regression) contribute to their superior performance. We also mention the role of the key predictors identified through permutation feature importance in driving these predictive outcomes.

**Comment 6**: Authors should pattern the motivation behind using this method to explain in the introduction.

**Response 6**: Thank you for your valuable comment. More explanation is added in the paragraph on line 108: “Using different machine learning methods in this context offers the promise of transforming traditional risk assessment and prognostication in cardiovascular health. Unlike conventional analytical techniques that often assume linearity or require strict parametric conditions, ML algorithms are capable of capturing complex, multifactorial interactions inherent in clinical data. This flexibility facilitates the integrative analysis of diverse data types, enhancing the potential to discover novel relationships that drive angina pectoris. Moreover, by applying techniques such as permutation feature importance, our approach not only identifies key predictors with high clinical relevance but also highlights potential targets for early intervention.”

**Comment 7**: There needs to be citation of recent papers on this topic and revise the literature section with slight Incorporation of recent ideas, for e.g.,

- A resource-aware multi-graph neural network for urban traffic flow prediction in multi-access edge computing systems
- Adaptive fuzzy backstepping secure control for incommensurate fractional order cyber–physical power systems under intermittent denial of service attacks
- Fuzzy adaptive control for consensus tracking in multiagent systems with incommensurate fractional-order dynamics: Application to power systems
- Revolutionizing E-Commerce With Consumer-Driven Energy-Efficient WSNs: A Multi-Characteristics Approach

**Response 7**: Thank you for your valuable comment regarding the literature coverage in our introduction. We have now revised the literature section and have strengthened it to incorporate recent advances and ideas (paragraph starting on line 86).

**Comment 8**: Could you elaborate on the hyperparameter optimization process for each machine learning algorithm?

**Response 8**: Thank you for this comment. More explanation is now added on hyperparameter tuning for each machine learning algorithm (Lines 269, 275, 282, 294, 300, 310, 317, and 323. There are also some details on line 326.

**Comment 9**: Was grid search or another optimization method used to fine-tune model parameters?

**Response 9**: Thank you for this question. Yes, it was. Hyperparameter tuning was carried out via the function RandomizedSearchCV from the scikit-learn library for all models except ANN, where the keras-tuner library v1.4.7 was used (Line 326). More details regarding the hyperparameter tuning settings for each model have been added.

**Comment 10**: Which features emerged as the most critical across different models, and how consistent were these findings?

**Response 10**: Thank you for highlighting the ambiguity regarding this issue. More explanation is now added on line 365, detailing the process of this technique. Noteworthy, uneven heartbeat consistently ranked first in all three models (Table 4, Line 409). Other prominent features included Normal activity dyspnea and Congestive heart failure. Paresthesia showed variability in importance, ranking second in Random Forest, third in Logistic Regression, and ninth in Linear Discriminant Analysis. Features such as Hospital admissions and “Any dyspnea” maintained high rankings. The inclusion of laboratory metrics like "Red blood cell count" and "Creatinine" also reflects the models' reliance on both clinical symptoms and laboratory values.

**Comment 11**: Did you observe any differences in feature importance when comparing the performance of individual classifiers versus ensemble models?

**Response 11**: Yes, we did: Two ensemble models were developed in this study (i.e. random forest and adaptive boosting), both of which used decision trees as the base model. Nonetheless, AdaBoost, as well as those individual classifiers not included in Table 4, did not perform well according to their AUC of ROC and this was reflected in high variability of their feature ranks. Therefore, we had not been able to draw reliable and meaningful results from those models to further enrich the findings.

**Comment 12**: Parameters of network have been enhanced using training data "until the model obtains the maximum accuracy". If this accuracy is the training accuracy, maybe over-fitting has been performed. If this accuracy is the testing accuracy, the system is adjusted over the same subset that is evaluated. A validation subset could be used to optimize the system with different data than the testing data and without performing over-fitting. In addition, it would be interesting to know which range of each parameter has been analyzed."?

**Response 12**: That is absolutely accurate. Thank you for spotting this error. The pertinent paragraph (paragraph on Line 338) erroneously describes the process which has been carried out. It is now revised. We monitored AUC while minimizing loss as calculated through binary cross-entropy. More importantly, the incorrect use of verbatim (cross-“validation”) would have misled the readers. To elucidate the details, 10-fold cross-validation has been performed while in each fold-split, the training set (80%) was further split into the train set and validation set (validation_split = 0.2). Therefore, each time the model was fitted, the sets were distributed as: train: 64%, val: 16%, test: 20%. The following is a setting in which the models were trained:

history = model.fit(xtrain, ytrain, epochs=10000, batch_size=64, validation_split=0.2, verbose=2, callbacks=[reduce_lr_loss, mcp_save, earlyStopping])

Therefore, the reported metrics include the performance of the models on the test sets (20% for each fold-split) and not the other 80% of the data. We have also used the early stopping method to prevent overfitting:

earlyStopping = EarlyStopping(monitor='val_loss', patience=100, verbose=0, mode='min')

We must again thank the esteemed reviewer for commenting on this issue. The methods section has now been revised regarding this process (paragraph starting on line 338).
