# Supplementary material for: Machine learning-based predictive modeling of angina pectoris in an elderly community-dwelling population: Results from the PoCOsteo study
Source: PLoS One. 2025 Aug 5;20(8):e0329023. doi: 10.1371/journal.pone.0329023 (PMC12324099; doi:10.1371/journal.pone.0329023)
Supplement: Attachment — Submitted filename: Response_to_Reviewers_auresp_2.docx [file pone.0329023.s003.docx]

**Reviewer #3**

Dear Reviewer,

Thank you for your thoughtful and constructive feedback on our revised manuscript titled “Machine learning-based predictive modeling of angina pectoris in an elderly community-dwelling population: results from the PoCOsteo study.” Your positive comments regarding our focus on angina pectoris prediction using a variety of machine learning methodologies are greatly valued. We are also grateful for your detailed suggestions for revisions, which we believe will enhance the clarity and robustness of our manuscript. In our response, we address each of your comments and outline the revisions made to improve the manuscript accordingly.

**Comment 1**: While the results are well-structured, it would enhance clarity if the authors specify confidence intervals along with AUC values for the top-performing models to better assess performance stability.

**Response 1**: We appreciate your suggestion to include confidence intervals alongside the AUC values for the top-performing models. We have now revised the results section to incorporate confidence intervals both on line 401 and figure 2.

**Comment 2**: Although the limitations are generally implied, an explicit limitations paragraph would strengthen the discussion. This could include the retrospective nature of the study, lack of external validation, or potential overfitting risks in ensemble models despite cross-validation.

**Response 2**: Thank you for your valuable feedback. We have now added a dedicated section that clearly outlines the “*limitations*” of our study (line 627) along with added explanation on line 638 through the end of the paragraph. The new section addresses the stated limitations. We appreciate your guidance in strengthening this aspect of our manuscript.

**Comment 3**: The permutation feature importance analysis reveals insightful predictors such as thrombotic vascular diseases and anemia. It would be valuable to comment briefly on how these findings align (or contrast) with existing literature on cardiovascular risk in the elderly.

**Response 3**: Thank you for this insightful comment. We have now added a brief discussion in the revised manuscript that contextualizes our findings on thrombotic vascular diseases (line 519), anemia (line 536), and renal failure (line 541) within the existing literature. Specifically, we reference studies that highlight the association between these conditions and increased cardiovascular risk, thereby reinforcing the relevance of our findings.

**Comment 4**: The manuscript is overall well-written, but a few minor typographical and grammatical adjustments may further improve readability. For instance, in the Abstract: "We aim to develop" may be better stated in the past tense ("We aimed to develop") given the retrospective design.

**Response 4**: We appreciate your helpful feedback regarding the clarity and readability of the manuscript. We have thoroughly reviewed the text and implemented several adjustments throughout the manuscript. We value your attention to detail, as these modifications contribute to the overall improvement of the manuscript.

**Comment 5**: While this is provided in response to editorial comments, ensure this data availability statement is also clearly reflected in the final manuscript version in the appropriate section.

**Response 5**: Thank you for your reminder regarding the data availability statement. We have now ensured that the data availability statement is clearly reflected (on line 668).

**Reviewer #4**

Dear Reviewer,

Thank you for your insightful and encouraging feedback on our manuscript. We are honored to have your expertise guiding our work and appreciate your recognition of the study's significance. Your questions regarding the rationale behind our chosen imputation methods, the integration of predictive models into clinical workflows, and the comparison of our results with recent studies in cardiovascular prediction are greatly helpful. We have carefully considered your suggestions and have made revisions to enhance the clarity and depth of our discussion. Additionally, we have addressed the minor grammatical refinements you recommended to improve the overall readability of the manuscript.

**Comment 1**: Can you provide detailed rationale and\or discussion on chosen imputation methods (SimpleImputer, mean/most frequent). Did alternative methods get considered, and if so why were they rejected?

**Response 1**: Thank you for your question regarding the imputation methods used in our study. We have added new explanation in the revised manuscript to clarify our choice of the SimpleImputer with mean and most frequent strategies on line 237: “… Implementing the second approach, we aimed for a method that balances simplicity and effectiveness. More complex methods such as KNN imputation or multiple imputation techniques, while potentially more accurate, also have certain drawbacks e.g., sensitivity to the choice of distance metrics and the number of neighbors or demanding more complex implementation and computation [31, 32]. Ultimately, the function used in the present analysis was SimpleImputer from the scikit-learn library [33], due to its straightforward implementation, computational efficiency, and the adequacy of its performance for our specific dataset. This choice strikes a balance between maintaining data integrity and ensuring the reliability of our analysis, while maintaining the overall distribution of the data and minimizing the introduction of bias that could arise from more complex imputation methods”.

**Comment 2**: Additional dedicated discussion on how your predictive models could be integrated into routine clinical workflows or decision-support systems may straighten up the final conclusion of your study.

**Response 2**: Thank you for your valuable suggestion. In response, we have now added a dedicated discussion in the revised manuscript that outlines potential pathways for implementing our models in clinical practice on line 613: “To effectively integrate our predictive models into routine clinical workflows and decision-support systems, it is essential to consider the practical application of these tools in everyday clinical settings. The models developed in this study, particularly the LR, LDA, and RF models, demonstrate not only strong predictive performance but also a level of interpretability that is crucial for clinical adoption. Instances include integrating these models into electronic health record systems to enable real-time risk assessments during patient evaluations, allowing clinicians to identify at-risk individuals promptly and suggest further assessments/interventions accordingly. Additionally, training programs for healthcare providers on how to utilize these predictive tools effectively can enhance their confidence in employing data-driven approaches in clinical practice. Ultimately, the successful integration of these models into clinical workflows has the potential to improve patient outcomes by enabling earlier detection and management of angina pectoris, thereby addressing a critical warning sign of underlying coronary heart disease in older adults”.

**Comment 3**: Also is it possible to strengthen the discussion by comparing your results more explicitly with recent similar studies involving ML models in cardiovascular prediction.

**Response 3**: We appreciate your insightful suggestion to enhance the discussion. In response, we have expanded the discussion section of the manuscript to include a more explicit comparison of our findings with relevant recent literature (paragraphs starting on lines 472 and 491). This includes highlighting similarities and differences in model performance, predictive features, and methodologies used in those studies. We appreciate your guidance in enhancing the depth of our discussion.

**Comment 4**: Minor grammatical refinements are recommended to enhance clarity and readability. An additional round of proofreading is suggested.

**Response 4**: Thank you for pointing out this issue. In response, we have conducted an additional round of proofreading to address these issues and ensure that the text is polished and clear. We have now made several changes throughout the manuscript.
